# Tracking Natal Dispersal in a Coastal Population of a Migratory Songbird Using Feather Stable Isotope (δ^2^H, δ^34^S) Tracers

**DOI:** 10.1371/journal.pone.0094437

**Published:** 2014-04-16

**Authors:** Samuel Haché, Keith A. Hobson, Erin M. Bayne, Steven L. Van Wilgenburg, Marc-André Villard

**Affiliations:** 1 Department of Biological Sciences, University of Alberta, Edmonton, AB, Canada; 2 Environment Canada, Saskatoon, SK, Canada; 3 Département de biologie, Université de Moncton, Moncton, NB, Canada; Liverpool John Moores University, United Kingdom

## Abstract

Adult birds tend to show high fidelity to their breeding territory or disperse over relatively short distances. Gene flow among avian populations is thus expected to occur primarily through natal dispersal. Although natal dispersal is a critical demographic process reflecting the area over which population dynamics take place, low recapture rates of birds breeding for the first time have limited our ability to reliably estimate dispersal rates and distances. Stable isotope approaches can elucidate origins of unmarked birds and so we generated year- and age-specific δ^2^H and δ^34^S feather isoscapes (ca. 180 000 km^2^) of coastal-breeding Ovenbirds (*Seiurus aurocapilla*) and used bivariate probability density functions to assign the likely natal areas of 35 males recruited as first-year breeders into a population located in northwestern New Brunswick, Canada. Most individuals (80–94% depending on the magnitude of an age correction factor used; i.e. 28–33 out of 35) were classified as residents (i.e. fledged within our study area) and estimated minimum dispersal distances of immigrants were between 40 and 240 km. Even when considering maximum dispersal distances, the likely origin of most first-year breeders was<200 km from our study area. Our method identified recruitment into our population from large geographic areas with relatively few samples whereas previous mark-recapture based methods have required orders of magnitude more individuals to describe dispersal at such geographic scales. Natal dispersal movements revealed here suggest the spatial scale over which many population processes are taking place and we suggest that conservation plans aiming to maintain populations of Ovenbirds and ecologically-similar species should consider management units within 100 or at most 200 km of target breeding populations.

## Introduction

Dispersal is a key component of animal demography because it controls rates of immigration and emigration among populations [1]–[3]. Most songbirds are characterized by low natal philopatry (but see [4]) and the rare data available suggest that first-year breeders may disperse over tens [5]–[7] or even hundreds of kilometres [8]–[11] prior to establishing their first breeding territory. Conversely, experienced breeders show high site fidelity [12]–[14]. Hence, gene flow and connectivity among avian populations should mainly reflect natal dispersal [15], which is commonly estimated as the straight-line distance moved by an individual from its natal area to its first breeding site [12]. Empirical estimates of natal dispersal distances are required to determine the spatial scale over which breeding populations of widely-distributed species interact and, thus, to define relevant conservation units. Data from mark recapture studies and indirect estimates based upon spatial correlations in abundance both suggest that movements likely occur over relatively short distances and that long-distance movements are rare (e.g. [5]–[7]). Unfortunately, such studies are generally inefficient for measuring long-distance dispersal ([16], but see [7]). Recently, intrinsic markers such as stable isotopes, trace elements, genetic markers, species assemblages of parasites, and diseases have been suggested as potential means by which dispersal movements can be tracked without marking individuals [16]. In particular, stable-hydrogen isotope ratios in feathers (δ^2^H_f_) have proven to be useful for detecting bird movements at continental scales [17].

Using δ^2^H_f_, Hobson et al. [9] obtained the first estimates of dispersal in Ovenbird (*Seiurus aurocapilla*) and American Redstart (*Setophaga ruticilla*) in western Canada based on the number of first-year birds that were “isotopic outliers” relative to expected baseline isotopic values for six study sites. Studds et al. [10] used this marker to generate a species-specific isoscape for Bicknell’s Thrush (*Catharus bicknelli*) and assignment tests to determine the area of likely origin of first-year breeders (i.e. second year individuals; hereafter SY). That study provided minimum natal dispersal distances and evidence for spatio-temporal discrepancy in demographic connectivity. Although single isotope approaches provide important insights into natal dispersal, it is generally recognized that multiple isotopes or combinations of techniques improves the spatial resolution for assigning individuals to their natal area (e.g. [18]–[21]). Stable isotope measurements of sulfur (δ^34^S) may be particularly useful in distinguishing individuals growing feathers in coastal regions vs. inland since marine-derived sulfates are generally more enriched in ^34^S than terrestrial sources and volatilized sulfates from ocean spray can be deposited inland over considerable distances directly or through precipitation [22]–[24].

We investigated the use of two stable isotope tracers to estimate natal dispersal distances in Ovenbird. Given that our study area was located within 160 km of the Gulf of St Lawrence, we jointly used δ^2^H_f_ and δ^34^S_f_ to obtain a finer-scale assignment than using δ^2^H_f_ alone. We anticipated significant longitudinal structure in δ^34^S_f_ based on distance from the coast and prevailing weather. We first created species-, age-, and year-specific feather isoscapes [25] for our study region and then assigned first-year breeders to their natal origin using these isoscapes and likelihood-based assignment tests based on bivariate probability density functions. Our specific objectives were to estimate the proportion of resident (hatched in the study area) vs. immigrant SY breeders recruited into our local breeding population and to determine the minimum distance moved by immigrants. Based on the typically low natal philopatry reported in songbirds, we predicted that most SY breeders would be immigrants. We had no *a priori* expectations regarding the spatial extent of natal dispersal movements by Ovenbirds as they have never been quantified. Most information available on distance of natal dispersal has been provided by mark-recapture studies. While mark-recapture studies are biased against detection of longer-distance dispersers, the isotope approach is biased against short-distance dispersers [9] since these will often be classified as residents. Nevertheless, an important objective of this study was to quantify the extent and frequency of long-distance natal dispersal to identify the area over which population dynamics of Ovenbirds take place.

## Methods

### Study Area

**Figure 1:**
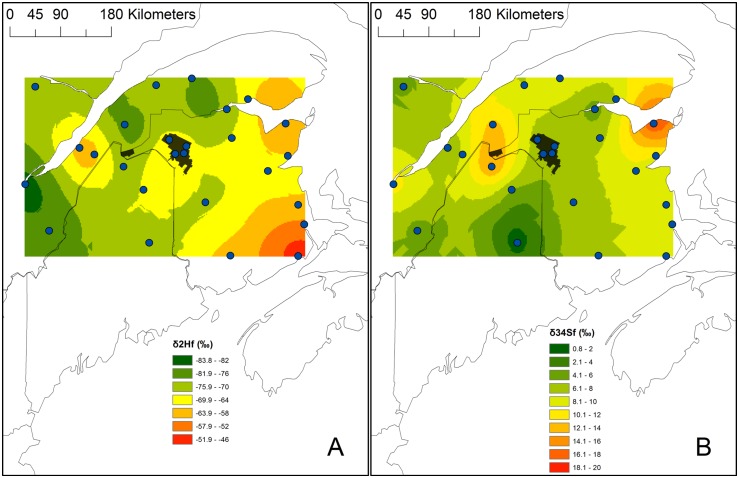
δ^2^H_f_ (A) and δ^34^S_f_ (B) Ovenbird isoscapes based on samples from ASY males collected in 2010 and calibrated 2011 and 2012 data. Isoscapes were based on ordinary Gaussian and ordinary spherical semivariogram models, respectively. Blue points are the 26 sampling locations and in black is the Black Brook district.

Since 2006, an individually-marked subpopulation of male Ovenbird has been monitored in northwestern New Brunswick, Canada (47°23′ N67°40′W; Black Brook district; Figure 1) to quantify the effects of an experimental harvest treatment on population dynamics [14], [26]. Black Brook is a 2000 km^2^ managed forest district owned by J.D. Irving Ltd. The landscape is a mosaic of spruce plantations and deciduous, mixedwood, and coniferous stands at the interface of the northern hardwoods and Acadian forest regions [27].

### Feather Samples, Benchmark Values, and Isoscapes

In 2010, most territorial males in the 250-ha area monitored (10×25 ha study plots; see [14] for details) that were not marked in previous breeding seasons (i.e. recruits) were captured (89%; 55/62) using mist-nets and playbacks of conspecific songs and marked with three color bands and a numbered aluminium band. Territorial individuals were also captured and marked in two additional 25-ha plots as part of a translocation experiment (see [28] for details; n = 12 and 16). Study plots were located in mature (81–160 years) and overmature (>161 years) deciduous stands that had not been altered in the past 30 years (n = 5) or managed through partial harvesting (n = 7; [14], [28]). Two 3^rd^ rectrices were plucked from each captured individual for aging and isotope analyses. Males were aged as SY or after second-year (ASY) individuals following the criteria of Donovan and Stanley [29] adapted by Bayne and Hobson [30]. In 2010–2012, we also collected feather samples from 48 returning ASY males banded in the previous year (hereafter “returning ASY”; n = 23, 14, and 11 for 2010, 2011, and 2012, respectively). During the same period, an additional 225 territorial males were captured within ca. 5 km of 22 additional locations outside the study area (10.2±1.4, mean ± SD, individuals per location; 16, 3, and 3 sampling locations in 2010, 2011, and 2012, respectively). Birds were captured in young (30–50 years), immature (51–80 years), and mature (81–160 years) deciduous and deciduous-dominated mixedwood forests. The total sampling area covered ca. 180 000 km^2^ (340×530 km; mean distance between closest sampling locations was 64.6 km, range = 28.9–134.0 km; Figure 1). Only feather samples from ASY males captured at these sampling locations were used to generate the feather isoscapes because they were believed to provide local values of δ^34^S_f_ and δ^2^H_f_ based on the assumption that ASY individuals have high breeding site fidelity [14], [31], [32] and moulting occurs in the vicinity of the breeding territory [33] (but see [34]). In total, we measured δ^2^H_f_ for all SY (2010) and returning ASY males from Black Brook (2010–2012), and for all ASY males from the 22 additional sampling locations (2010–2012). Values of δ^34^S_f_ were also measured for all SY males (2010) from Black Brook, but only for returning ASY males from 2010 and 2012, owing to logistical constraints. For the same reason, only three individuals per additional sampling location were analyzed for δ^34^S_f_ (n = 66).

Stable-hydrogen isotope ratios from feathers collected in the same breeding area can vary among years [35], [36]. As our goal was to assign SY males captured in 2010 (feathers grown in 2009) to their natal area the previous year, we controlled for a potential yearly variation in isotope values by creating a year-specific δ^2^H_f_ isoscape reflecting expected values for feathers grown in 2009. Thus, we only used δ^2^H_f_ values from returning ASY males (Black Brook) captured in 2010 (feathers grown in 2009) as the benchmark values for the year-specific δ^2^H_f_ isoscape. We could not visit our entire study region in a single year to create a year-isoscape and so applied a correction factor for δ^2^H_f_ values from samples collected at locations surrounding Black Brook in 2011 (feathers grown in 2010; n = 3) and 2012 (feathers grown in 2011; n = 3) based on the yearly variation we saw in ASY males at Black Brook. Specifically, we compared mean δ^2^H_f_ values from returning ASY males (Black Brook) captured in 2010 vs. 2011 and 2010 vs. 2012. We then used these differences to adjust δ^2^H_f_ from ASY males captured at sampling locations outside Black Brook in 2011 and 2012. Only δ^2^H_f_ values from six sampling locations had to be calibrated as the ASY males from the remaining 16 locations were all captured in 2010. This approach assumes that year-to-year variation in δ^2^H from growing-season precipitation is similar across the total sampling area. Values of δ^34^S_f_ were not calibrated to account for annual variation as there was no *a priori* reason to expect annual variation in this isotope. However, we still tested for a year effect using a *t*-test.

Mean δ^34^S_f_ and δ^2^H_f_ from individuals captured at the 12 study sites in Black Brook were pooled in 4 locations (3 sites per location) covering the extent of the study area (Figure 1). Sites within each of these locations were 3–6 km apart and each location was separated by at least 7 km (see also [14], [28]). Study sites were pooled to provide a similar number of ASY males for each of the 26 sampling locations used to generate feather isoscapes. Mean values from all sampling locations were assigned a centroid and used to generate the isoscapes. Spatial autocorrelation among mean values from each sampling location was modelled for both isotopes using semivariance analyses and kriging interpolations. We investigated several semivariogram models (ordinary spherical, circular, exponential, Gaussian, or linear), and selected the model that minimized the root-mean-square error. Isoscapes were created in ArcGIS 10 using the Geostatistical Analyst extension (ESRI, Redlands, CA).

Research was approved by the Université de Moncton Animal Care and Use Committee (Permit Numbers: 10-06, 11-04, and 12-02), Canadian Wildlife Service (Permit Numbers: SC2710 and SC2751), and U.S. Fish and Wildlife Service (Permit Number: MB11009A-0).

### Stable Isotope Analyses

Surface oils were removed from feathers using a 2∶1 chloroform:methanol solution. Samples were prepared according to [37] and analyses were conducted at the Stable Isotope Hydrology and Ecology Laboratory of Environment Canada (δ^34^S_f_ in 2010–2012 and δ^2^H_f_ in 2010 and 2012) and the Colorado Plateau Stable Isotope Laboratory (δ^2^H_f_ in 2011). High-temperature (1350°C) flash pyrolysis generating an H_2_ pulse for each sample (350±20 μg) was used to obtain δ^2^H_f_ measurements by continuous-flow isotope-ratio mass spectrometry (CF-IRMS). To account for exchangeable hydrogen in keratins, comparative equilibration was done using in-house keratin working standards (BWB = −108‰, CFS = −147.7‰, and CHS = −187‰; [38]). The two laboratories used the same protocol and standards for δ^2^H_f_ measurements. Values of δ^34^S_f_ were also measured by CF-IRMS (3500±100 μg of tissue per sample). All results are expressed as nonexchangeable deuterium (δ^2^H_f_) and sulphur (δ^34^S_f_) isotopic ratios in units of per mil (‰) and normalized to Vienna Standard Mean Ocean Water – Standard Light Antarctic Precipitation (VSMOW-SLAP) and Canyon Diablo Triolite, respectively. Based on within-run analyses of keratin standards, we assumed measurement error to be ca. ±2‰ for both δ^2^H_f_ and δ^34^S_f_.

### Assignment Test

Previous studies have reported differences in δ^2^H_f_ between juvenile (i.e. nestlings, hatch-year, or SY birds) and ASY individuals growing feathers at the same site ranging between 6–32.6‰ [10], [36], [39]. Our goal was to assign SY males assumed to have grown their rectrices mostly during the first few weeks postfledging (i.e. rectrices only start emerging from feather sheaths a few days prior to fledging; S.H., MAV, and EMB, pers. obs.). Thus, to link SY results to an ASY feather isoscape, a correction factor to account for the documented effect of age was required. Based on a long-term study, Studds et al. [10] reported that δ^2^H_f_ was on average ∼6‰ lower in first-year breeders/fledglings than in adult Bicknell’s Thrushes at the northern edge of their study area. This portion of the Bicknell’s Thrush range (i.e. southern Quebec, New Brunswick, and Gaspe Peninsula) corresponded to the extent of our isoscapes and this comparison based on values from first-year breeders/fledglings is more appropriate than values from nestling Ovenbirds obtained by Haché et al. [36]. Indeed, it is unclear whether comparisons between δ^2^H_f_ from growing (nestlings) versus inert (first-year breeders/fledglings) tissue provide appropriate age correction factors. Also, the Bicknell’s thrush is expected to be of the same guild as the Ovenbird and hence should make a reasonable surrogate species. Hobson et al. [40] found no evidence for an age effect (hatching- and second-year vs. ASY) on δ^2^H_f_. Although we believe that the most parsimonious approach was to use a range of age correction factors from no correction to adding 6‰ to all SY males [10], [40], we generated assignment tests based on five different age correction factors (+6‰, +3‰, no correction, –3‰, and –6‰) to examine how sensitive the results of assignment tests were to a range of correction factors. The correction factor was applied to δ^2^H_f_ of each SY male prior to assigning their origin to the ASY feather isoscapes.

We generated spatially-explicit assignment tests using bivariate normal probability density functions (BNPDF; [41]) to determine the likely origin of SY males. The likelihood that a given raster cell (*j*; 2 km×2 km cell size) within the feather isoscapes was a potential origin was estimated based on the following equation:
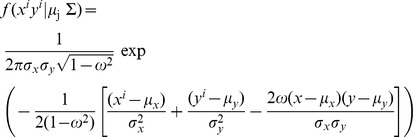
Where *f(x^i^y* ^i^|*μ_j
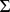
_*) is the likelihood that an individual (*i*) with given δ^2^H_f_ (*x^i^*) and δ^34^S_f_ (*y^i^*) values originated from a raster cell *j*, while μ is the mean, σ is the standard deviation, and *ω* is the correlation between δ^2^H_f_ (*x*) and δ^34^S_f_ (*y*) predicted for each raster cell (*j*) within the feather isoscapes. Predicted mean isotopic composition for each raster cell (*j*) was generated from the calibrated isoscapes (δ^2^H_f_ and δ^34^S_f_). The parameter μ*_j_* is therefore a vector of means for each raster cell (*j*) within the feather isoscapes presented as:



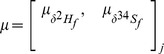



Each BNPDF was parameterized using both δ^2^H_f_ and δ^34^S_f_ isoscapes and the following variance-covariance matrix:
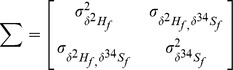
where σ^2^δ^2^H_f_ and σ^2^δ^34^S_f_ are the expected variance for each isotope ratio and σδ^2^H_f_, σδ^34^S_f_ is the covariance between the pair of isotope ratios. The variance-covariance matrix was validated to define the likelihood a cell within the extent of our isoscape corresponded to a potential origin. This was achieved by using a subset of 66 samples from ASY males (i.e. the three ASY males from each of the 22 sampling location outside Black Brook with values for both δ^2^H_f_ and δ^34^S_f_; 36% of all ASY in the dataset). We assumed a spatial stationarity of variance-covariance across our study area [41]. Standard deviations representing variation within sampling location were 10.5‰ and 3.8‰ for δ^2^H_f_ and δ^34^S_f_, respectively, and the correlation coefficient from the covariance was 0.29. For all SY males (2010), we generated a binary raster layer to classify each cell as a likely (1) or unlikely (0) origin based on a 2∶1 odds ratio (i.e. “1” was assigned to cells that were consistent with the upper 67% of the cumulative probabilities and “0” was assigned to all other cells). Odds ratios between 2∶1 and 4∶1 provided the best depiction of the geographic distribution of assigned versus true origins for samples from multiple individuals ([21] on advantages of using 2∶1 odds ratio). We estimated the likely origin of all SY males by summing the binary surfaces. We conducted similar analyses to assign the 23 returning ASY males captured in 2010 (Black Brook) to the isoscapes to determine the spatial resolution and classification accuracy (i.e. percent of returning ASY with an assigned area that overlapped their known origin) provided by this approach. To compare predictions from results of models using different odds ratio, we also generated assignment tests for all SY males using a 4∶1 odds ratio and two age correction factors (±6‰). Lastly, we used the predicted δ^2^H_f_ and δ^34^S_f_ from our isoscape for the four sampling locations within Black Brook (Figure 1) and generated fictional individuals for which their origin was considered “unknown”. We assigned their likely area of origin to the bivariate isoscape and calculated the minimum detectable dispersal distance as the perpendicular distance from a sampling location to the closest area assigned as unlikely origin. This value reflected the minimum distance from which an individual could have been assigned as immigrant. This is not a universal minimum detectable dispersal distance and only reflects the minimum detectable distance given our isoscapes and the location of our study site within those isoscapes. Assignment tests were generated using scripts employing the raster [42] package in R v 2.15.2. For each SY male assigned as an immigrant (i.e. assigned origin did not overlap the sampling location where it was captured; as opposed to a resident), we calculated the minimum linear distance between breeding location and natal area [10] using ArcGIS v 10.

## Results

**Figure 2:**
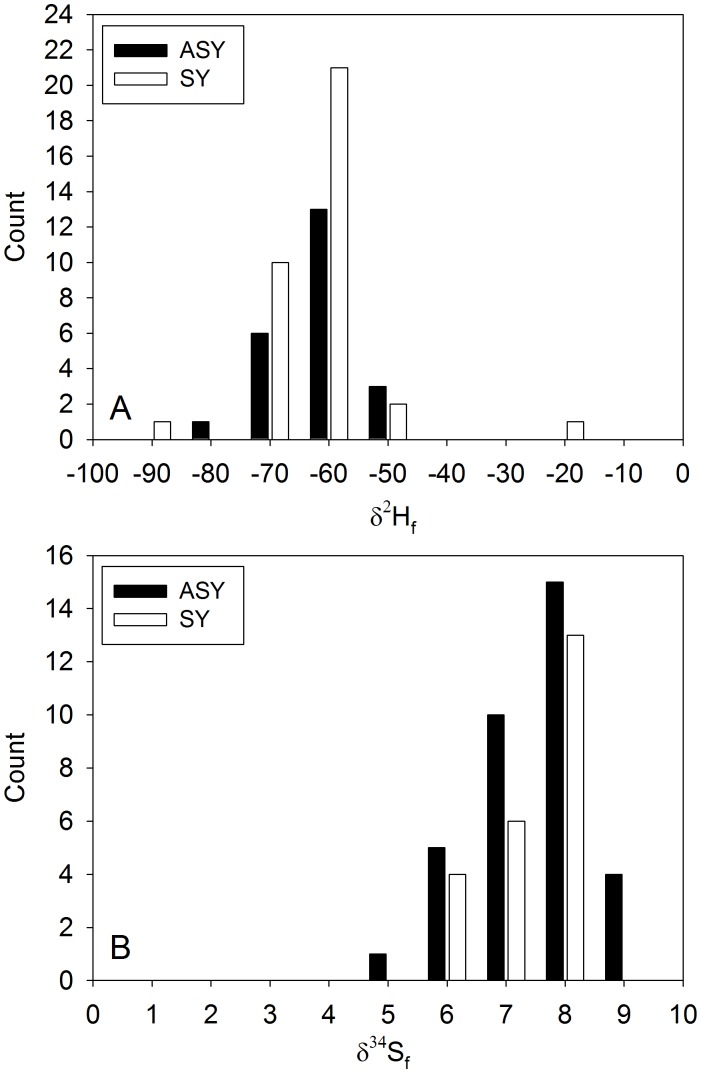
Histograms of δ^2^H_f_ (A) and δ^34^S_f_ (B) from returning ASY (n = 23) and SY (n = 35) males breeding in the Black Brook district, New Brunswick, in 2010.

Of the 10.2 male Ovenbirds captured on average (±1.4, SD; n = 225) at each of the 22 sampling locations outside Black Brook, 6.5 were ASY (±2.1; range of 3–10 individuals per location; n = 142; 63%). When added to the 23 ASY males captured in Black Brook in 2009 and subsequently recaptured in 2010 (5.8±2.2 ASY per sampling location), samples from a total of 165 and 89 ASY males were used to produce the δ^2^H_f_ and δ^34^S_f_ isoscapes, respectively (Figures 1–2). In Black Brook, we also recaptured 14 returning ASY males in 2011 and 11 in 2012. There was year-to-year variation in δ^2^H_f_ from returning ASY males (2010 = −67.0±5.3‰, 2011 = −88.1±4.1‰, and 2012 = −78.9±5.1‰; see also [36]). Thus, to correct for this year effect in δ^2^H_f_, we added 21.1‰ to the δ^2^H_f_ of 20 ASY males captured at sampling locations (n = 3) outside Black Brook in 2011 and 11.9‰ to those of 25 ASY males captured at the three other sampling locations in 2012. Mean δ^34^S_f_ values differed significantly between years (2010 = 6.79±0.58‰ and 2012 = 6.30±0.29‰; t_32_ = −2.6, p = 0.014), but this difference was negligible when considering the 2‰ measurement error. In 2010, we captured 35 SY males breeding in Black Brook (δ^2^H_f_ = −68.0±10.2‰ and δ^34^S_f_ = 7.06±0.86‰; Figure 2).

The δ^2^H_f_ isoscape was generated using ordinary kriging based upon a Gaussian semivariogram model (root-square-mean error = 4.59). The model-estimated parameters were a major range of 120.7 km, nugget of 0.81, and sill of 31.8. Data for δ^34^S_f_ were kriged to create an isoscape based upon a spherical semivariogram model (root-square-mean error = 1.03). The semivariogram model parameters for the δ^34^S_f_ isoscape included a major range of 120.7 km, nugget of 0, and sill of 11.8. The δ^2^H_f_ isoscape showed generally more enriched values in southeastern portion of the isoscape (∼50‰) with gradual depletion along a southeastern-northwestern gradient, whereas the δ^34^S_f_ isoscape corresponded to the anticipated coastal effect with more enriched values mostly along the coast (10–20‰) creating an east-west gradient (Figure 1).

**Figure 3:**
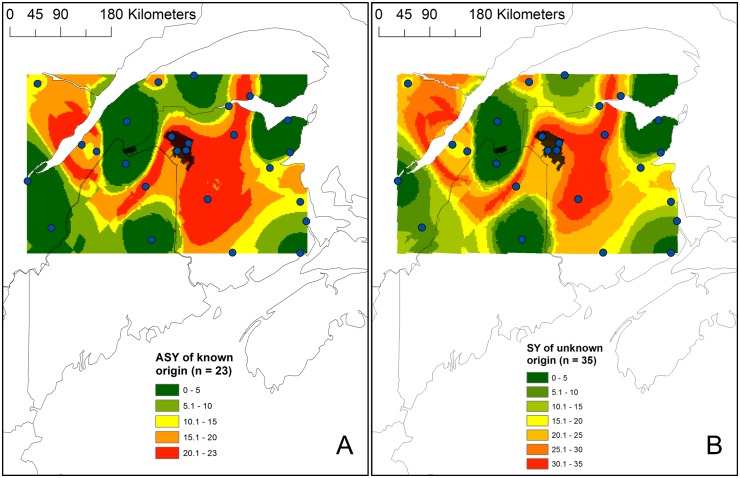
Geographic distribution of the assigned origin of 23 returning ASY (A) and 35 SY (B) male Ovenbirds known to have bred in the Black Brook district (in black), New Brunswick. Assignments of each male to the δ^2^H_f_ and δ^34^S isoscapes were based on using bivariate normal probability density functions and no δ^2^H_f_ age correction factor (2∶1 odds ratio). Maps represent the sum of all binary raster layers of each individual assignment. Blue points are the 26 sampling locations and in black is the Black Brook district.

The assigned origin of 22 of the 23 ASY males of known origin overlapped their sampling location (classification accuracy of 95.6%; Figure 3A). Similarly, 94.3% of the SY males (33/35) were classified as residents under four of the five correction factors (no correction, −3‰, +3‰, and +6‰; 2∶1 odds ratio; Figures 3B, S1B, and S2). The two individuals classified as an immigrants had minimum dispersal distances ranging from 40–54 km and 221–237 km (Table S1). The assignments based on a −6‰ correction factor had 80% of the SY males (28/35; Figure S1A) classified as residents. Using this correction factor, the seven immigrants had minimum dispersal distances ranging from 23–210 km, but only one individual had a minimum dispersal distance>61 km (Table S1). The minimum detectable dispersal distance of our four sampling locations in Black Brook was 40±14 km (Figure S3). Using a 4∶1 odd ratios provided more conservative estimates with only one and two individuals classified as immigrants under the +6‰ and −6‰ age correction factors, respectively (Table S1; Figures S4).

The maximum distance of dispersal (i.e. maximum distance of likely origin) of SY males could not be estimated because in all cases, it reached the edge of the isoscape, suggesting that they could have originated from an area beyond our defined feather isoscape. However, the population-level assigned origin (i.e. sum of the binary surfaces for each SY male) indicated that likely origins of SY males only reached edges of the isoscapes in small areas in the southeast and northeast, suggesting that the majority of SY males tended to have a maximum natal dispersal distance<200 km from Black Brook (Figures 3B, S1B, and S2). Under the −6‰ age correction factors, a larger proportion of SY males might have originated beyond the northwestern and southwestern edges of the isoscape (Figure S1A). This effect was even greater when we used a 4∶1 odds ratio with a −6‰ age correction factor (Figure S4A). Interestingly, this edge effect was not as important for assignment tests based on a 4∶1 odds ratio and +6‰ age correction factor (Figure S4B). The assigned area of returning ASY males (Figure 3A) tended to be similar to that of SY males based on four of the six age correction factors (no correction, –3‰, +3‰, and +6‰), suggesting that the maximum natal dispersal distance is a conservative estimate as it corresponds to the maximum spatial resolution provided by this approach.

## Discussion

By combining age- and year-specific δ^34^S_f_ and δ^2^H_f_ Ovenbird isoscapes and bivariate assignment tests along with the most parsimonious age correction factors (i.e. no correction, +3‰, and +6‰), we showed that most (94%) SY males recruited in our coastal-breeding population likely originated locally (i.e. hatched in our 2000 km^2^ study area; residents). Given that stable isotope analyses have been shown to be better-suited to identify long-distance dispersal than mark-recapture studies, this finding was unexpected based on the low natal philopatry generally reported in songbirds [12]. However, our range of dispersal distances (40–200 km) did overlap with those provided by mark recapture studies and indirect estimates based upon time-lagged, pairwise correlations in abundance (10–100 km; e.g. [5]–[7]). When summing the assigned areas of all SY males, our estimates of maximum distances of natal dispersal still suggested that movements generally occurred within 200 km (Figure 3B and S2). This information on natal dispersal movements offers insight into the area over which many population processes are taking place and, ultimately, how populations are structured. Hence, our approach can be used to identify the spatial scale at which conservation plans for the Ovenbird and ecologically-similar songbirds should be implemented (see also [6], [15]).

Estimates of minimum natal dispersal distances suggest that most SY males can be considered as residents and, in the rare instances where individuals were classified as immigrants (n = 2 based on the most parsimonious age correction factors), immigrants would have originated within 240 km of our study area. The Black Brook district covers 2000 km^2^ (ca. 80 km× 25 km), suggesting that a high proportion of males recruited into the district’s Ovenbird population likely hatched “locally”. Based on δ^2^H_f_ outliers, Hobson et al. [9] considered 9.5% (±8.1%; n = 6) of SY male Ovenbirds breeding in western Canada as long-distance dispersers which is consistent with the percentage of immigrants (5.7%) in our study. However, they could not separate short-distance dispersers from residents. A band-recovery study from Europe [8] and a North American study using one-year time-lagged correlations in Breeding Bird Survey data [6] both estimated mean natal dispersal distances<100 km for several landbird species. Alternatively, Studds et al. [10] showed that minimal natal dispersal movements in Bicknell’s Thrush reached 700 km and they considered that 59% of first-year breeders were immigrants. Unlike the Ovenbird, Bicknell’s Thrush occurs at low abundance, population sizes are declining [43], and they have a small, highly-patchy breeding range [44]. However, the study sites surveyed by Studds et al. [10] might be smaller than ours (Black Brook, ca. 2000 km^2^) and spatial scale must be considered when making such comparisons. Nonetheless, Paradis et al. [8] showed that interspecific variation in natal dispersal distance was negatively correlated with population size and the spatial extent of the breeding range, consistent with the observed differences between Ovenbird and Bicknell’s Thrush populations. In contrast, Prothonotary Warbler (Protonotaria citrea) and Tree Swallow (*Tachycineta bicolor*) also have different population sizes and breeding ranges [45], [46], yet show similar in natal dispersal distances. Median natal dispersal distance in Tree Swallow is 2.3 km (1.3% of individuals dispersed 50–210 km; [5]), as opposed to 1.4 km (maximum of 17 km) in Prothonotary Warbler [7]. However, those results are from nestbox studies and inferences from such studies to populations breeding in natural nest sites may be misleading [47], [48]. Those results were also from mark recapture studies and although those authors obtained dispersal distance distributions, their approaches cannot be used to estimate emigration rates unless we account for sampling effort. Using intrinsic markers such as stable isotopes results in every capture being a recapture [16] and provides unbiased estimates of immigration rates given the spatial resolution provided by this approach (40 km in this study).

The assigned origin of all SY males reached the edge of our isoscape, limiting our ability to generate accurate estimates of maximum natal dispersal distances. This was especially the case when using a −6‰ age correction factor and assignment tests based on a 4∶1 odds ratio. However, when using the most parsimonious age correction factors, the likely origin only reached smaller areas at the edge of the isoscape (ca. 190 km from Black Brook; Figure 3B and S2), suggesting that we likely captured most of the area that would have been assigned had we examined isoscapes of larger spatial extent. This is based on the assumption that similar isotopic composition is unlikely to occur over large areas beyond the extent of our isoscape as a result of the latitudinal and longitudinal gradients reported for δ^34^S_f_ and δ^2^H_f_, respectively. Similar coastal conditions at the same latitude also do not occur elsewhere in the Ovenbird’s breeding range [49]. Nevertheless, we recognize that dual isotopic mapping over the entire range of the species would be required to consider all possible isotopic origins of birds arriving in our study area. Also, the assigned likely natal origin of the SY males monitored in this study (Figure 3B) covered an area similar to the assigned origin of the returning ASY males from Black Brook (known origin; Figure 3A) suggesting that our estimates of maximum distance of natal dispersal are constrained by the spatial resolution (i.e. uncertainty) provided by this approach and might even be shorter. Given the spatial resolution provided, it is also possible that some individuals classified as residents might have immigrated over short distances and were within the minimum range of detectability using the isotope approach. This support results from previous studies suggesting that stable isotope analyses would have limited ability to quantify short-distance dispersal (e.g. [9]). Lastly, the classification accuracy of ASY of known origin was 95.6% and 94% (33/35) of SY males were considered resident. Hence, it is unclear whether the two immigrants are indeed immigrants or this pattern was a result of the classification accuracy from our bivariate approach.

We minimized sources of error in our δ^2^H_f_ isoscape by controlling for interspecific, age, and year effects which are all known to influence δ^2^H_f_ [36], [39], [40]. Elevation is another factor influencing δ^2^H_f_ [50], [51]. Most of the area covered by our isoscapes varied from 0–600 m in elevation [52], but we are confident that our sampling design integrated the majority of this variation within our δ^2^H_f_ isoscape. Other potential factors explaining spatial variation in the δ^2^H_f_ isoscape are fine-scale variation in temperature, moisture level, and amount of precipitation [53] which might be especially important near coastlines (see also[34]).

Unlike δ^2^H_f_, we had no *a priori* reason to expect inter-annual variation in δ^34^S_f_. Similarly, δ^34^S_f_ is unlikely to be influenced by the age of an individual because little diet-tissue isotopic discrimination occurs in these isotopes as a result of metabolic activity [54], [55]. Overall, the spatial variation observed in the δ^34^S_f_ isoscape corresponds to the anticipated coastal effect (deposition of volatilized marine sulfates) previously observed [22]–[24], [56], [57]. However, in some instances, the overall gradient in δ^34^S_f_ across our isoscape as a function of distance to the ocean seems to have been altered by local processes that we do not fully understand (reviewed by [22], [54], [58]).

We calibrated δ^2^H_f_ of SY males based on age-related variation in δ^2^H_f_ provided by Hobson et al. [40] and a 10-year study on Bicknell’s Thrush [10]. The assumption that this range in the correction factor (0–6‰) is consistent among songbird species and breeding seasons needs to be validated as it could have important implications when assigning origin of SY males to ASY feather isoscapes. Only subtle differences were detected between these results and the assigned origin of SY males based on −3‰ and −6‰ correction factors (Table S1). It has often been suggested that 2∶1 odds ratio would offer the most reasonable likelihood of correct versus incorrect assignments of individuals to geographic origin (reviewed by [21]), but this remains a relatively subjective threshold and we believe that is important to compare these results from those more conservative odd ratios (e.g. 4∶1 and 9∶1).

Low recapture rates often prevent the use of extrinsic markers to evaluate natal dispersal. The main advantage of the isotope approach is that it allows tracking natal dispersal of a completely random sample of a population of first-year breeders, whereas mark-recapture studies require an initial marked population that can be biased. Multivariate assignment tests have been used to assign the geographic origin of numerous taxa (e.g. birds [59], [60], invertebrates [61], [62], and mammals [63]), but most studies documented migratory patterns and fewer explored age-specific dispersal movements. Our results are consistent with those from other studies suggesting that although philopatry is low in songbirds, natal dispersal typically occurs over relatively short distances. Findings from this study could be used to test predictions about effects of habitat fragmentation on immigration rates and dispersal movements (e.g. [64]). However, future studies should consider: 1) using larger isoscapes, i.e. whole breeding range, to provide estimates of both minimum and maximum dispersal distances; 2) investigating natal dispersal in females; and 3) including additional chemical markers (e.g. δ^13^C, δ^15^N, ^87^Sr/^86^Sr, and trace elements; [65]–[67]) to determine the maximum spatial resolution available to investigate biologically relevant spatial scales for management planning to conserve bird populations. Nonetheless, we recommend that to conserve Ovenbird populations and those from ecologically-similar songbird species, the relevant demographic unit, i.e. the area of origin of potential recruits, corresponds to a radius of 100 or at most 200 kilometers (see also [68], [69]).

## Supporting Information

Figure S1 **Geographic distribution of the assigned origin of 35 SY male Ovenbirds known to have bred in the Black Brook district, New Brunswick.** Assignments of SY males to the δ^2^H_f_ and δ^34^S isoscapes were based on using bivariate normal probability density functions and −6‰ (A) and −3‰ (B) δ^2^H_f_ age correction factors (2∶1 odds ratio). Maps represent the sum of all binary raster layers of each individual assignment. Blue points are the 26 sampling locations and in black is the Black Brook district.
(TIF)

Figure S2 **Geographic distribution of the assigned origin of 35 SY male Ovenbirds known to have bred in the Black Brook district, New Brunswick.** Assignments of SY males to the δ^2^H_f_ and δ^34^S isoscapes were based on using bivariate normal probability density functions and +3‰ (A) and +6‰ (B) δ^2^H_f_ age correction factors (2∶1 odds ratio). Maps represent the sum of all binary raster layers of each individual assignment. Blue points are the 26 sampling locations and in black is the Black Brook district.
(TIF)

Figure S3 **Minimum detectable dispersal distance (in grey) for individuals captured at one of the four sampling locations (in blue) within the Black Brook district (in black).** Predicted δ^2^H_f_ and δ^34^S_f_ from our isoscapes were used for the four sampling locations within the Black Brook district to generate fictional individuals for which their origin was considered “unknown”. The likely area of origin was assigned to the bivariate isoscape and we calculated the minimum detectable dispersal distance as the perpendicular distance from a sampling location to the closest area assigned as unlikely origin (0).
(TIF)

Figure S4 **Geographic distribution of the assigned origin of 35 SY male Ovenbirds known to have bred in the Black Brook district, New Brunswick.** Assignments of SY males to the δ^2^H_f_ and δ^34^S isoscapes were based on using bivariate normal probability density functions and –6‰ (A) and +6‰ (B) δ^2^H_f_ correction factors (4∶1 odds ratio). Maps represent the sum of all binary raster layers of each individual assignment. Blue points are the 26 sampling locations and in black is the Black Brook district.
(TIF)

Table S1 **SY male Ovenbirds (n = 35) classified as resident (1) or immigrant (0) depending on whether or not the assigned area of origin overlapped the study area (Black Brook).** Assignment tests were based on 5 different age correction factors. In parentheses is the minimum distance of dispersal for each immigrant. Unless indicated otherwise (4∶1), assignment tests were based on a 2∶1 odds ratio. ID represents individual birds.
(DOCX)
