# Supplementary figures and images for: Tracking Natal Dispersal in a Coastal Population of a Migratory Songbird Using Feather Stable Isotope (δ2H, δ34S) Tracers
Source: PLoS One. 2014 Apr 16;9(4):e94437. doi: 10.1371/journal.pone.0094437 (PMC3989223; doi:10.1371/journal.pone.0094437)

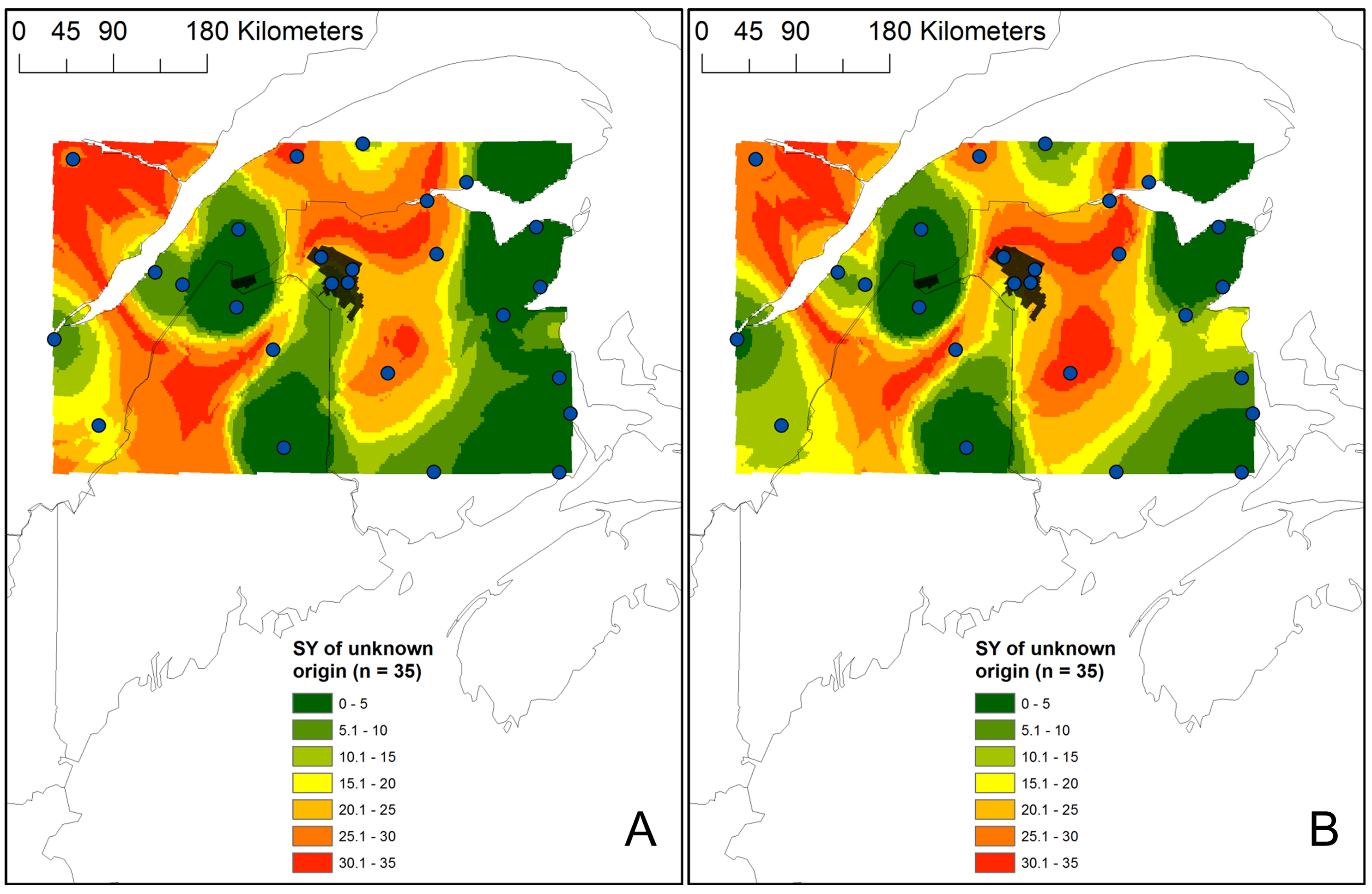

Supplement: Figure S1 — Geographic distribution of the assigned origin of 35 SY male Ovenbirds known to have bred in the Black Brook district, New Brunswick. Assignments of SY males to the δ2Hf and δ34S isoscapes were based on using bivariate normal probability density functions and −6‰ (A) and −3‰ (B) δ2Hf age correction factors (2∶1 odds ratio). Maps represent the sum of all binary raster layers of each individual assignment. Blue points are the 26 sampling locations and in black is the Black Brook district. (TIF) [file pone.0094437.s001.tif]

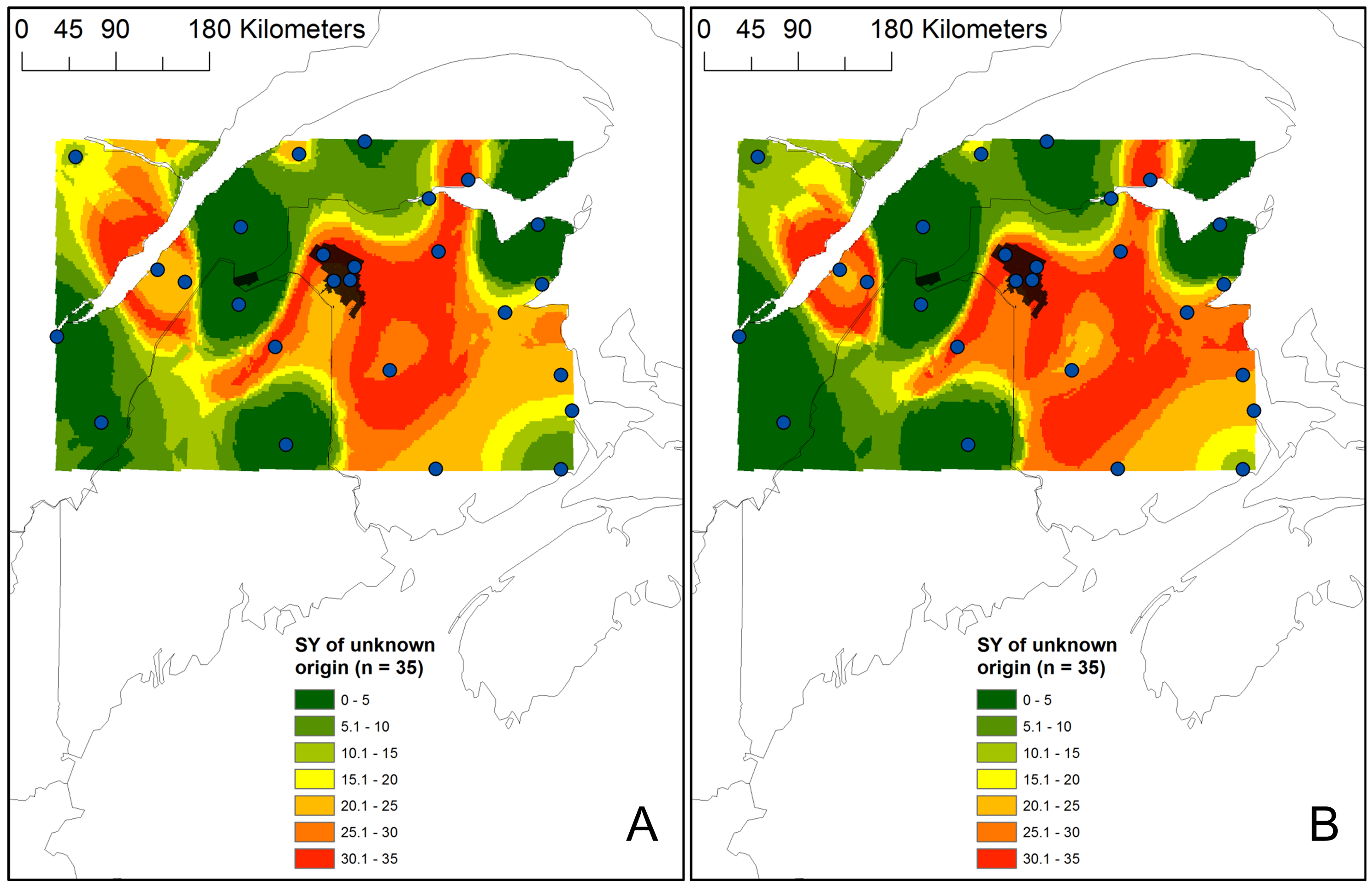

Supplement: Figure S2 — Geographic distribution of the assigned origin of 35 SY male Ovenbirds known to have bred in the Black Brook district, New Brunswick. Assignments of SY males to the δ2Hf and δ34S isoscapes were based on using bivariate normal probability density functions and +3‰ (A) and +6‰ (B) δ2Hf age correction factors (2∶1 odds ratio). Maps represent the sum of all binary raster layers of each individual assignment. Blue points are the 26 sampling locations and in black is the Black Brook district. (TIF) [file pone.0094437.s002.tif]

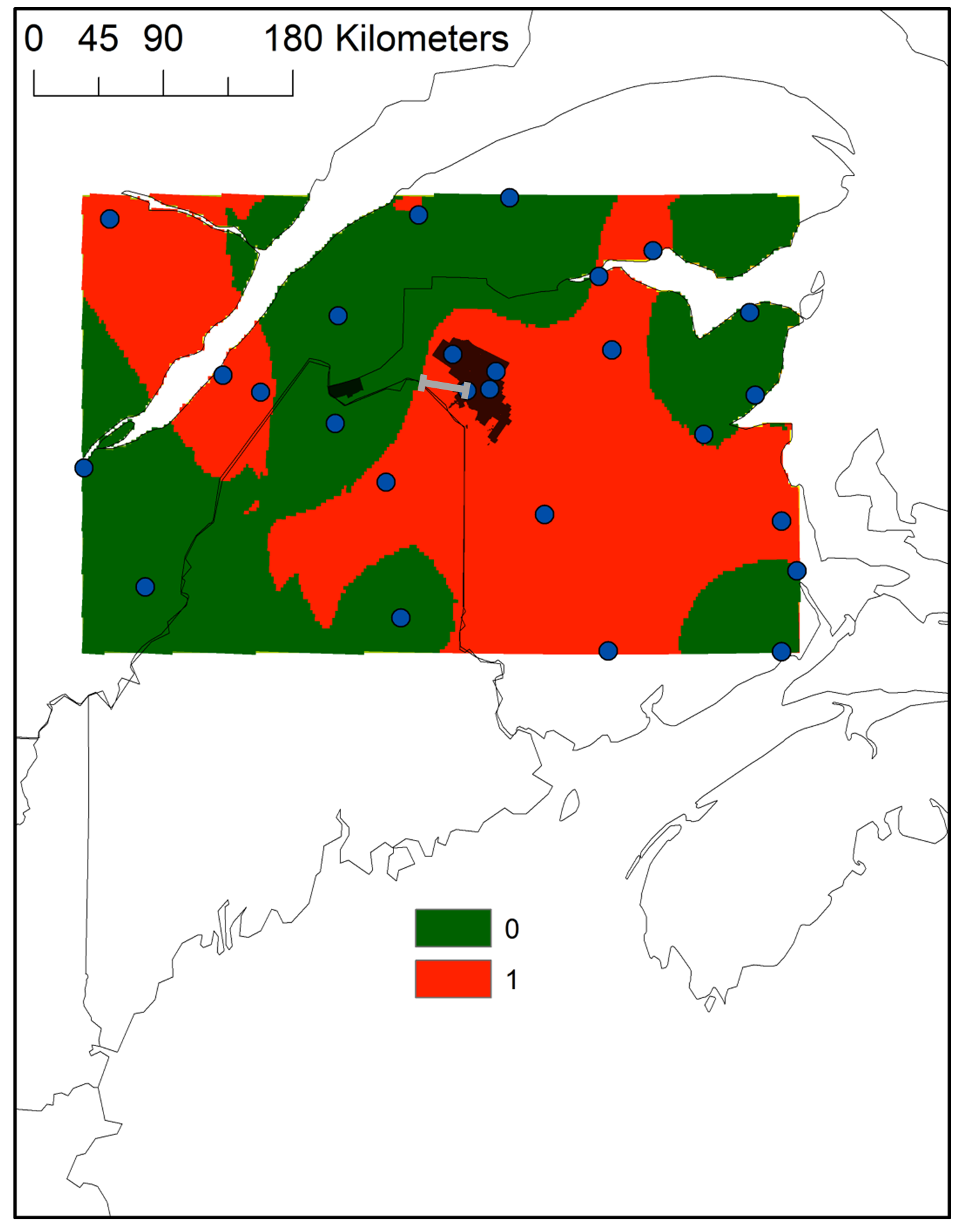

Supplement: Figure S3 — Minimum detectable dispersal distance (in grey) for individuals captured at one of the four sampling locations (in blue) within the Black Brook district (in black). Predicted δ2Hf and δ34Sf from our isoscapes were used for the four sampling locations within the Black Brook district to generate fictional individuals for which their origin was considered “unknown”. The likely area of origin was assigned to the bivariate isoscape and we calculated the minimum detectable dispersal distance as the perpendicular distance from a sampling location to the closest area assigned as unlikely origin (0). (TIF) [file pone.0094437.s003.tif]

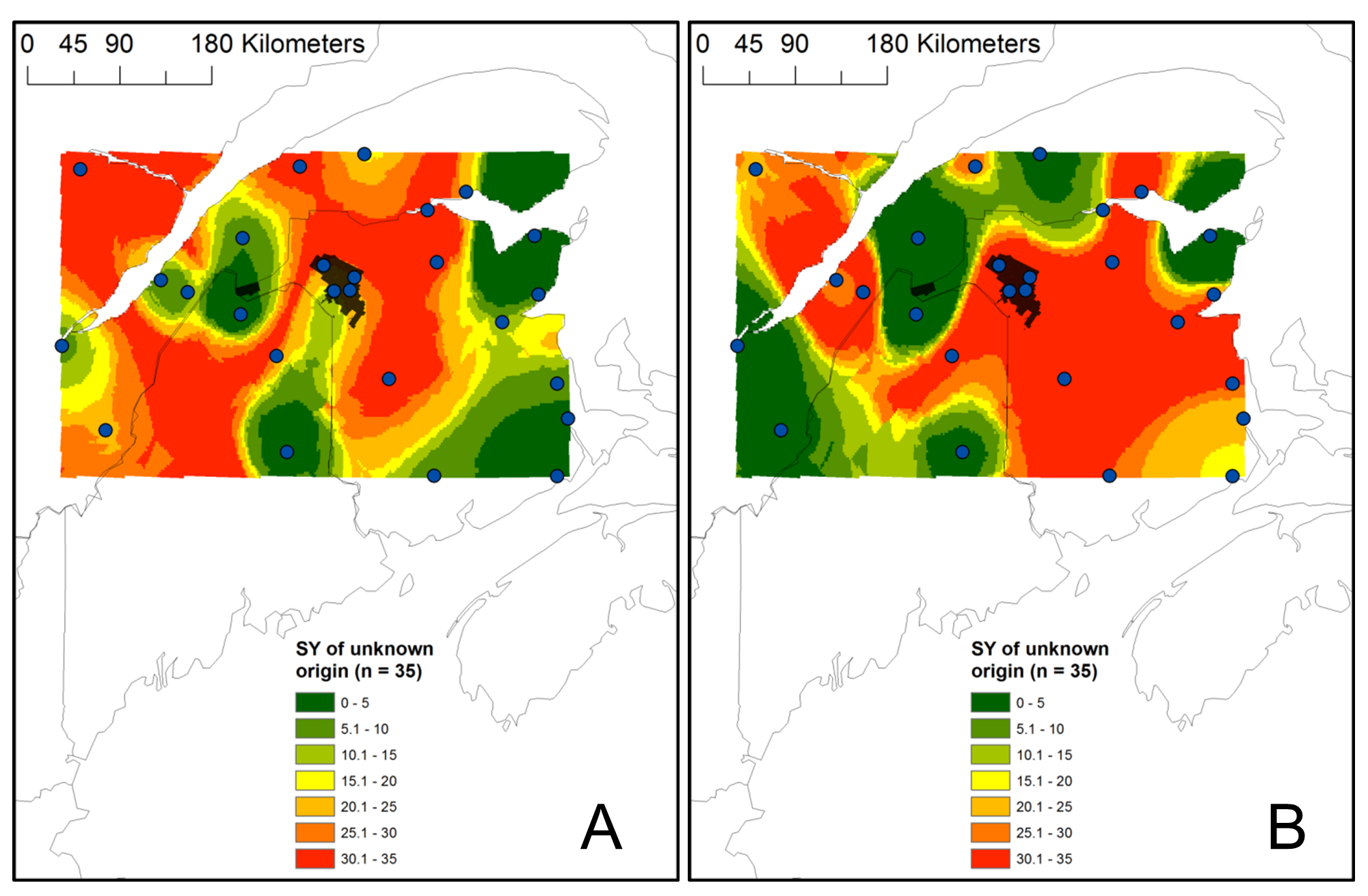

Supplement: Figure S4 — Geographic distribution of the assigned origin of 35 SY male Ovenbirds known to have bred in the Black Brook district, New Brunswick. Assignments of SY males to the δ2Hf and δ34S isoscapes were based on using bivariate normal probability density functions and –6‰ (A) and +6‰ (B) δ2Hf correction factors (4∶1 odds ratio). Maps represent the sum of all binary raster layers of each individual assignment. Blue points are the 26 sampling locations and in black is the Black Brook district. (TIF) [file pone.0094437.s004.tif]
