# Supplementary material for: Tracking Natal Dispersal in a Coastal Population of a Migratory Songbird Using Feather Stable Isotope (δ2H, δ34S) Tracers
Source: PLoS One. 2014 Apr 16;9(4):e94437. doi: 10.1371/journal.pone.0094437 (PMC3989223; doi:10.1371/journal.pone.0094437)
Supplement: Table S1 — SY male Ovenbirds (n = 35) classified as resident (1) or immigrant (0) depending on whether or not the assigned area of origin overlapped the study area (Black Brook). Assignment tests were based on 5 different age correction factors. In parentheses is the minimum distance of dispersal for each immigrant. Unless indicated otherwise (4∶1), assignment tests were based on a 2∶1 odds ratio. ID represents individual birds. (DOCX) [file pone.0094437.s005.docx]

| ID | | Site | | - 6‰ | | - 3‰ | | No calibration | | + 3‰ | | + 6‰ | | - 6‰ (4:1) | | + 6‰ (4:1) | |
| --- | --- | --- | --- | --- | --- | --- | --- | --- | --- | --- | --- | --- | --- | --- | --- | --- | --- |
| 1 | | 1 | | 0 (39.1) | | 1 | | 1 | | 1 | | 1 | | 1 | | 1 | |
| 2 | | 1 | | 1 | | 1 | | 1 | | 1 | | 1 | | 1 | | 1 | |
| 3 | | 1 | | 1 | | 1 | | 1 | | 1 | | 1 | | 1 | | 1 | |
| 4 | | 1 | | 1 | | 1 | | 1 | | 1 | | 1 | | 1 | | 1 | |
| 5 | | 1 | | 1 | | 1 | | 1 | | 1 | | 1 | | 1 | | 1 | |
| 6 | | 1 | | 0 (36.4) | | 1 | | 1 | | 1 | | 1 | | 1 | | 1 | |
| 7 | | 1 | | 1 | | 1 | | 1 | | 1 | | 1 | | 1 | | 1 | |
| 8 | | 1 | | 1 | | 1 | | 1 | | 1 | | 1 | | 1 | | 1 | |
| 9 | | 2 | | 1 | | 1 | | 1 | | 1 | | 1 | | 1 | | 1 | |
| 10 | | 2 | | 1 | | 1 | | 1 | | 1 | | 1 | | 1 | | 1 | |
| 11 | | 2 | | 1 | | 1 | | 1 | | 1 | | 1 | | 1 | | 1 | |
| 12 | | 2 | | 1 | | 1 | | 1 | | 1 | | 1 | | 1 | | 1 | |
| 13 | | 2 | | 1 | | 1 | | 1 | | 1 | | 1 | | 1 | | 1 | |
| 14 | | 2 | | 0 (60.9) | | 0 (53.6) | | 0 (48.7) | | 0 (43.8) | | 0 (39.7) | | 0 (46.9) | | 1 | |
| 15 | | 2 | | 1 | | 1 | | 1 | | 1 | | 1 | | 1 | | 1 | |
| 16 | | 2 | | 1 | | 1 | | 1 | | 1 | | 1 | | 1 | | 1 | |
| 17 | | 2 | | 0 (34.6) | | 1 | | 1 | | 1 | | 1 | | 1 | | 1 | |
| 18 | 3 | | 1 | | 1 | | 1 | | 1 | | 1 | | 1 | | 1 | |  |
| 19 | 3 | | 0 (210.4) | | 0 (221.3) | | 0 (229.1) | | 0 (232.8) | | 0 (236.9) | | 0 (163.8) | | 0 (223.7) | |  |
| 20 | 3 | | 1 | | 1 | | 1 | | 1 | | 1 | | 1 | | 1 | |  |
| 21 | 3 | | 0 (23.2) | | 1 | | 1 | | 1 | | 1 | | 1 | | 1 | |  |
| 22 | 3 | | 1 | | 1 | | 1 | | 1 | | 1 | | 1 | | 1 | |  |

| ID | Site | - 6‰ | -3‰ | No calibration | + 3‰ | + 6‰ | - 6‰ (4:1) | + 6‰ (4:1) |
| --- | --- | --- | --- | --- | --- | --- | --- | --- |
| 23 | 3 | 1 | 1 | 1 | 1 | 1 | 1 | 1 |
| 24 | 3 | 1 | 1 | 1 | 1 | 1 | 1 | 1 |
| 25 | 3 | 1 | 1 | 1 | 1 | 1 | 1 | 1 |
| 26 | 3 | 1 | 1 | 1 | 1 | 1 | 1 | 1 |
| 27 | 3 | 0 (22.6) | 1 | 1 | 1 | 1 | 1 | 1 |
| 28 | 3 | 1 | 1 | 1 | 1 | 1 | 1 | 1 |
| 29 | 3 | 1 | 1 | 1 | 1 | 1 | 1 | 1 |
| 30 | 4 | 1 | 1 | 1 | 1 | 1 | 1 | 1 |
| 31 | 4 | 1 | 1 | 1 | 1 | 1 | 1 | 1 |
| 32 | 4 | 1 | 1 | 1 | 1 | 1 | 1 | 1 |
| 33 | 4 | 1 | 1 | 1 | 1 | 1 | 1 | 1 |
| 34 | 4 | 1 | 1 | 1 | 1 | 1 | 1 | 1 |
| 35 | 4 | 1 | 1 | 1 | 1 | 1 | 1 | 1 |
| Total |  | 28 | 33 | 33 | 33 | 33 | 33 | 34 |
